# Antibiotic Guideline Adherence at the Emergency Department: A Descriptive Study from a Country with a Restrictive Antibiotic Policy

**DOI:** 10.3390/antibiotics12121680

**Published:** 2023-11-29

**Authors:** Mariana B. Cartuliares, Sara N. Søgaard, Flemming S. Rosenvinge, Christian B. Mogensen, Mathias Amdi Hertz, Helene Skjøt-Arkil

**Affiliations:** 1 Department of Emergency Medicine, University Hospital of Southern Denmark, 6200 Aabenraa, Denmark; mbc@rsyd.dk (M.B.C.);; 2 Department of Regional Health Research, University of Southern Denmark, 6200 Aabenraa, Denmark; 3 Department of Clinical Microbiology, Odense University Hospital, 5000 Odense, Denmark; 4 Research Unit of Clinical Microbiology, Department of Clinical Research, University of Southern Denmark, 5000 Odense, Denmark; 5 Department of Infectious Diseases, Odense University Hospital, University of Southern Denmark, 5000 Odense, Denmark; 6 Research Unit of Infectious Diseases, Department of Clinical Research, University of Southern, 5000 Odense, Denmark

**Keywords:** antibiotics, emergency department, community-acquired pneumonia, urine tract infection

## Abstract

Background: Denmark has a low level of antimicrobial resistance (AMR). Patients hospitalized with suspected infection often present with unspecific symptoms. This challenges the physician between using narrow-spectrum antibiotics in accordance with guidelines or broad-spectrum antibiotics to compensate for diagnostic uncertainty. The aim of this study was to investigate adherence to a restrictive antibiotic guideline for the most common infection in emergency departments (EDs), namely community-acquired pneumonia (CAP). Method: This multicenter descriptive cross-sectional study included adults admitted to Danish EDs with a suspected infection. Data were collected prospectively from medical records. Results: We included 954 patients in the analysis. The most prescribed antibiotics were penicillin with beta-lactamase inhibitor at 4 h (307 (32.2%)), 48 h (289 (30.3%)), and day 5 after admission (218 (22.9%)). The empirical antibiotic treatment guidelines for CAP were followed for 126 (31.3%) of the CAP patients. At 4 h, antibiotics were administered intravenously to 244 (60.7%) of the CAP patients. At day 5, 218 (54.4%) received oral antibiotics. Conclusion: Adherence to CAP guidelines was poor. In a country with a restrictive antibiotic policy, infections are commonly treated with broad-spectrum antibiotics against recommendations.

## 1. Introduction

Antimicrobial resistance (AMR) is a global threat requiring urgent action. Five million people lose their lives annually to infections with drug-resistant bacteria [1]. The misuse and overuse of antibiotics contribute to the development of multi-resistant bacteria [2] and are associated with extended hospital stays, greater costs, and increased mortality [3]. Denmark has one of the lowest prevalences of AMR in the world [1] due to the restrictive use of antibiotics [4]. But even in Denmark, AMR is increasing, and every 20th patient admitted to a Danish emergency department (ED) is colonized with multi-resistant bacteria [5]. To improve antibiotic practices in hospitals, the Danish Health Authority has issued national recommendations [6] which aim to reduce the use of carbapenems, cephalosporins, and fluoroquinolones (CCF antibiotics) as much as possible. To avoid intravenous (IV) catheter-related infections and extended hospital stays, a shift to oral treatment is recommended as soon as the patient is stable. These recommendations are implemented in regional antibiotic stewardship guidelines [7].

Patients presenting at EDs with infectious diseases are commonly diagnosed with community-acquired pneumonia (CAP) [8,9]. The CAP diagnosis is primarily based on clinical symptoms (e.g., cough, sputum, fever, and chest pain), combined with imprecise diagnostic tools: the X-ray of the lungs, blood tests, and microbiological analyses of sputum samples [10]. Patients admitted with CAP can present with unspecific symptoms and elderly patients often present with vague or unspecific symptoms such as confusion or delirium [11,12]. Furthermore, different comorbidities may affect the clinical presentation [13]. This leaves physicians with a challenging choice: adhere to guidelines or treat with broad-spectrum antibiotics in an attempt to compensate for diagnostic uncertainty.

The aim of this study was to investigate the adherence to regional CAP guidelines at Danish EDs. The objectives were:

(1) To describe antibiotic prescriptions at the EDs;
(2) To investigate adherence to empirical antibiotic treatment guidelines for CAP upon acute admission;
(3) To investigate whether the IV treatment of CAP is switched to oral treatment during an acute admission.

## 2. Materials and Methods

### 2.1. Study Design and Setting

This descriptive cross-sectional study was based on data from INDEED (Infectious Diseases in Emergency Departments), a prospective multifaceted multi-center study. The protocol for the INDEED study has been published previously [14]. We included patients admitted to an ED at Odense University Hospital, Hospital Lillebaelt in Kolding, and Hospital Sønderjylland in Sønderborg and Aabenraa between 1 March 2021 and 28 February 2022. The four hospitals had a total catchment population of approximately 775,000 people. The study was reported in accordance with the STROBE (Strengthening the Reporting of Observational Studies in Epidemiology) statement [15].

### 2.2. Participants

Adults (≥18 years) admitted to the ED with suspected infection were consecutively screened during daytime and evenings on weekdays and invited to participate in the study by project assistants. The treating physician allocated patients to one of two diagnosis-related groups depending on the suspected focus of infection: community-acquired pneumonia (CAP) or “other infections”. We focused on patients with suspected CAP. Only patients able to give informed consent were included in the study.

We excluded patients directly transferred to intensive care, admitted within the last 14 days, with verified COVID-19 infection within the previous 14 days, with severe immunodeficiency, or if participation delayed life-saving treatment. Further information on participant eligibility is described in the study protocol [14].

### 2.3. Data Source and Variables

We extracted data from the patients’ electronic medical records, including age, sex, microbiological analysis of sputum and blood, CURB-65 (confusion, uremia, respiratory rate, blood pressure [16]), Danish Emergency Process Triage (DEPT) [17], fever (temperature ≥ 38 °C) [18], C-reactive protein (CRP) [19], and the type and route of administration of antibiotics at three different time points after admission: 4 h, 48 h, and 5 days.

Antibiotics were categorized into six groups:

(1) Narrow-spectrum beta-lactamase sensitive penicillin (Therapeutic Chemical Code (ATC J01CE);
(2) Extended-spectrum beta-lactamase penicillin (ATC J01CA);
(3) Penicillin with beta-lactamase inhibitor (ATC J01CR);
(4) CCF antibiotics (ATC J01DB, J01DC, J01DD, J01DE, J01DH, and J01MA);
(5) Macrolides (ATC J01A);
(6) Others (aminoglycosides (ATC J01G), trimethoprim (ATC J01EA01), short-acting sulfonamides (ATC J01EB), the combination of sulfamethoxazol and trimethoprim (ATC J01EE01), nitrofurantoin (ATC J01XE01), tetracyclines (ATC J01AA), vancomycin (ATC A07AA09, J01XA01), cloxacilin (ATC J01CF), lincosamider (ATC J01F), and metronidazole (ATC P01AB01, J01XD01).

In Danish EDs, initial antibiotic treatment is started after a tentative diagnosis, often within the first 4 h of admittance. Regional guidelines for empirical antibiotic treatment are shown in Table 1. Flowcharts based on clinical guidelines for the management of CAP based on the CURB-65 score are presented in the Supplementary Materials of Figure S1 (CURB-65 < 3) and Figure S2 (CURB-65 ≥ 3).

Cefuroxime and/or a macrolide was considered in accordance with guidelines if the patient was registered as allergic to penicillin. According to regional guidelines, a review of all the started antibiotic treatments is mandatory at 48 h and every third day thereafter. The review must include the type of antibiotic, dosage, route of administration, and duration. Furthermore, the review should be based on clinical response, microbiological results, and additional diagnostic workup [7].

### 2.4. Statistical Methods

Descriptive analyses were performed. Data on categorical or binary variables were presented as numbers (n) and proportions (%), and data on continuous variables for non-normal distribution were summarized in median and interquartile ranges (IQR). Data showing the six groups of antibiotics, route of administration (IV and oral), and time of prescription (4 h, 48 h, and day 5) were described in numbers (n) and percentages (%) and presented graphically with a margins plot. For data analysis we used Stata Statistical Software: Release 17. College Station, TX, USA: StataCorp LLC.

## 3. Results

We included 966 patients, 12 were excluded after inclusion, due to a positive COVID-19 test, leaving 954 patients with suspected infection in the analysis. Among these, 402 (42.1%) were suspected of CAP (Table 2). Information about the prescribed antibiotics was missing for one patient at 48 h and two on day 5.

### 3.1. Antibiotic Prescriptions

#### 3.1.1. Antibiotic Prescription for Patients Suspected of Infection

Penicillin with beta-lactamase inhibitor was the most commonly prescribed antibiotics at 4 h (307 patients, 32.2%), 48 h (289 patients, 30.3%), and day 5 (218, 22.9%). Narrow-spectrum beta-lactamase sensitive penicillin was prescribed to 237 patients (24.8%) at 4 h and 216 (22.6%) at 48 h. Antibiotics were not prescribed to 226 patients (23.7%) at 4 h, 205 (21.5%) at 48 h, and 259 (27.2%) at day 5. The use of macrolides, CCF antibiotics, and other antibiotics was between 10.2 and 10.7% at all times. The numbers and percentages are shown in Table S1 in the Supplementary Materials.

#### 3.1.2. Antibiotic Prescription for Patients Suspected of CAP

**Figure 1:**
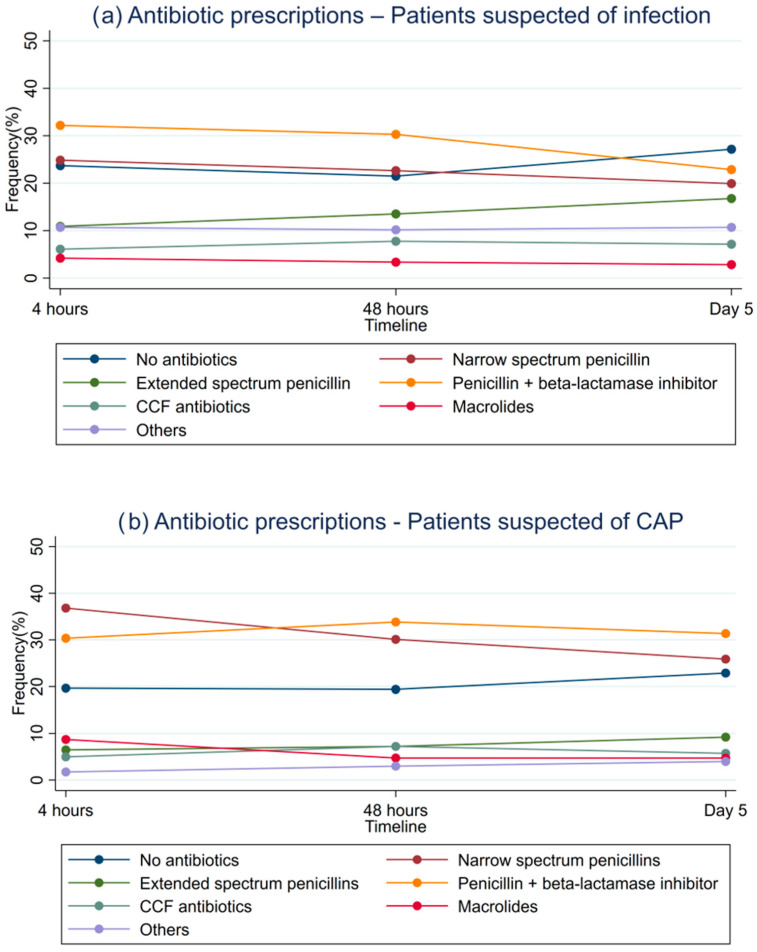
Antibiotic prescriptions at 4 h, 48 h, and day 5 for patients admitted with suspected infection (**a**) and suspected community-acquired pneumonia (CAP), and CFF (carbapenems, cephalosporins, and fluoroquinolones) (**b**).

At 4 h after admission, 147 (36.6%) patients had narrow-spectrum beta-lactamase sensitive penicillin prescribed, 122 (30.3%) had penicillin with beta-lactamase inhibitor, and antibiotics were not prescribed to 79 (19.2%) (Figure 1b). Five days after admission, penicillin with beta-lactamase inhibitor (126 (31.3%)) was prescribed 25% more often than narrow-spectrum beta-lactamase sensitive penicillin (101 (25.1%)).

### 3.2. Adherence to Empirical Antibiotic CAP Treatment Guidelines

Guidelines for empirical treatment were followed in 126 (31.3%) patients suspected of CAP. Among the patients suspected to have CAP, 53 (13.5%) had a CURB-65-score ≥ 3, indicating that most of the included patients suspected of CAP should have been prescribed narrow-spectrum antibiotics.

### 3.3. Route of Administration of Antibiotic Treatment of CAP

**Figure 2:**
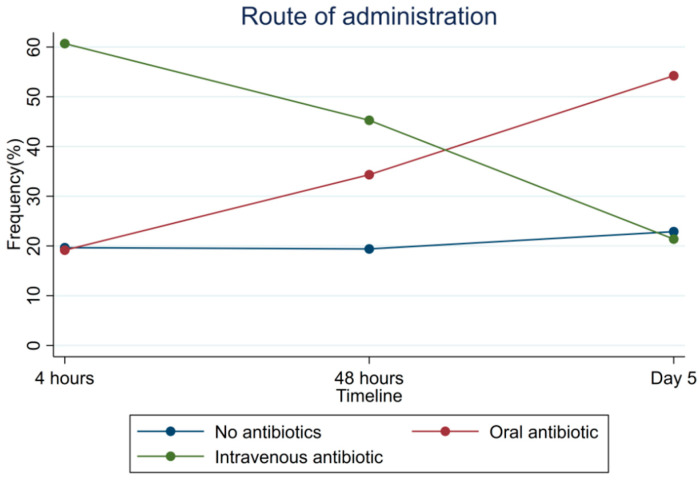
Route of administration for patients admitted suspected of CAP: intravenous antibiotics, oral antibiotics, and no antibiotics at 4 h, 48 h, and day 5.

IV and oral antibiotics administration at the three time points (4 h, 48 h, and day 5) during the first five days for patients suspected of CAP are illustrated in Figure 2 and Supplementary Materials Table S1. At 4 h, 244 patients (60.7%) were administered IV antibiotics, decreasing to 182 (45.3%) at 48 h and 86 (21.4%) on day 5. The administration of oral antibiotics showed the opposite pattern; 77 (19.2%) at 4 h, 138 (34.3%) at 48 h, and 218 (54.4%) on day 5. At all three time points, the number of patients not treated with antibiotics varied between 19.4% and 22.9% and less than 1.2% were treated with a combination of oral and IV antibiotics.

## 4. Discussion

Antibiotics were prescribed to approximately three-quarters of the patients acutely admitted with suspected infection. Overall, the most prescribed treatment for CAP was penicillin with beta-lactamase inhibitor. Only 126 (31.3%) of the CAP patients were treated in accordance with regional guideline. Most patients were treated with IV antibiotics at 4 h and three-quarters of the patients received oral antibiotic at day 5.

Antibiotic prescriptions in Denmark have been reported as among the lowest in Europe [20]. It is therefore remarkable that the prescription rate in our study was higher than that in a US multicenter study [21]. A likely explanation is that the study populations were different—in terms of both type and severity of illness. In our study, patients were older and more patients received antibiotics before admission.

In general, Denmark has a prudent use of antibiotic and a low AMR. However, our study found that broad-spectrum antibiotics often were prescribed for mild and moderate CAP, where narrow-spectrum antibiotics were recommended. In line with guideline recommendations for urinary tract infections or abdominal infections, acutely admitted patients suspected of infections other than CAP were prescribed relatively broad-spectrum antibiotics [7]. Furthermore, it is likely that patients without a clear site of infection and/or unspecific clinical symptoms may have received broad-spectrum antibiotics to account for diagnostic uncertainty [22].

We took penicillin allergy into account. However, we know from clinical practice and previous studies that only 10–20% of patients reporting a history of penicillin allergy are truly allergic [23]. Therefore, the adherence to guidelines might be even lower if adjusted for truly penicillin allergy.

When patients are admitted to the ED, the physician has to assess the patients and start treatment within a few hours. Our findings reveal a trend in Danish EDs where the guidelines are often overruled despite the presence of a clear regional and national antibiotic stewardship program. A study conducted in the Netherlands emphasizes that adherence to treatment guidelines is significantly influenced by the healthcare providers responsible for treating patients [24]. Furthermore, a systematic review from the US found that atypical manifestations in patients are a significant contributor to misdiagnosis or diagnostic errors [25]. Therefore, both the personal preferences of healthcare providers and the patients’ presentation could be an explanation for the low adherence to guidelines we found in Danish EDs.

In the EDs, high work pressure and patients with complex symptoms and comorbidities could affect the physician’s decision to overrule the guidelines and treat the patient with broad-spectrum antibiotics due to insecurity regarding prognosis, symptoms, or diagnosis. Factors affecting the physician’s antibiotic prescription were investigated in a study from the UK, where they found that the decision mainly depended on concerns about adverse consequences and beliefs [26]. This is supported by a Dutch study, where physicians may choose to continue IV administration due to concerns about safety [24].

In our study, and in line with our guideline, patients were often switched from IV to oral antibiotics within the first five days [27], indicating that they were aware of the guidelines and had fewer barriers preventing adherence than when prescribing empirical antibiotics [28,29]. A Dutch study observed a similar switch rate for patients treated for CAP [30]. An American study reported that a focus on clinical guidelines, recommending an early switch from IV to oral antibiotics, lowered the number of CAP patients hospitalized three days after admission by 6% [31]. Besides reducing catheter-related infections, the benefits of oral treatment are reduced nursing time, length of stay, treatment costs, increased patient satisfaction, and faster discharge [32,33,34,35].

In our study, we equated the inclusion allocation with the tentative diagnosis, which formed the basis for antibiotic prescription. In a proportion of cases, the patients were included and allocated after the primary evaluation but before the physician had access to blood tests, urine tests, and X-rays to rely on for the tentative diagnosis. This resulted in a mismatch between the tentative diagnosis and the allocation of inclusion. There would be no difference between the inclusion allocation and the tentative diagnosis for patients who underwent a first evaluation, the admission assessment, and the 4 h evaluation simultaneously. Our study did not indicate the extent to which multiple examinations were intertwined, and therefore, we could not account for this. We acknowledge that this was a weakness of our study.

A strength of our study is the prospective multicenter set-up with almost complete data on antibiotic treatment. In addition, exactly the same antibiotic guideline was used in the included EDs throughout the period. Our paper describes a pragmatic study conducted in a country with low levels of AMR and restrictive guidelines for antibiotic treatment. The insights and findings from this study can be generalized to other EDs in similar settings.

We only included patients who could give informed written consent and we excluded patients with cognitive impairments and the most severely ill patients. If these patients had been included, it is probable that more patients would have been treated with broad-spectrum antibiotics. However, this would likely be in accordance with guidelines and improve adherence. Infection control measures to contain the COVID-19 pandemic reduced the prevalence of several other common respiratory pathogens during the study period and may have resulted in a relatively atypical prescription pattern.

Our findings indicate that antimicrobial stewardship programs in Denmark should focus on prescription patterns in the EDs in order to increase guideline adherence and reduce AMR. Future interventions such as the improved and faster diagnostics of infections could contribute to the more prudent use of antibiotics. The use of point-of-care tests, such as the detection of respiratory pathogens with polymerase chain reaction [36], urine flow cytometry [37], or biomarkers [38] at the ED, has the potential to minimize the empiric use of broad-spectrum antibiotics.

It may enable physicians in EDs to target the initial antibiotic treatment or even withhold antibiotics for some patients.

## 5. Conclusions

This pragmatic study from a country with low AMR and restrictive antibiotic guidelines showed only 31% adherence to guidelines when prescribing initial empirical antibiotics to patients suspected of CAP. This indicates the need for greater attention to antibiotic prescription practices in the ED. In contrast, two out of three patients had switched to oral antibiotics by day five, indicating better guideline implementation and adherence.

## Figures and Tables

**Table 1:** Empirical clinical guidelines for patients with admitted suspected CAP.

Severity of CAP	First Choice	Penicillin Allergy	Therapy Duration (iv * and Oral)
CURB-65: < 3 (Mild)	Benzylpenicillin or Phenoxymethylpenicillin	Cefuroxime or Macrolide	5 days
CURB-65 ≥ 3 (Moderate-severe)	Benzylpenicillinllin + Macrolide †	Cefuroxime + Macrolide	7 days
CURB-65 ≥ 3 ** (Severe)	Piperacillin-tazobactam + Macrolide	Cefuroxime + Macrolide	7 days

* Intravenous route. † Macrolide: The treatment is only extended if PCR is positive for *Legionella pneumophila*, *Mycoplasma pneumoniae,* or *Chlamydophila pneumoniae*. ** CURB-65 ≥ 3: radiological involvement of multiple lung lobes, or hypoxia with O_2_ saturation < 92%, or sepsis.

**Table 2:** Characteristics of patients admitted to the emergency departments with suspected infection.

Characteristics	Patients Suspected of Infection	Patients Suspected of CAP	Missings
Total n (%)	954 (100.0)	402 (42.1)	n (%)
Age, median (IQR)	73.0 (59.0; 81.0)	74.0 (62.0; 82.0)	0/(0.0)
Sex, male, n (%)	513 (53.8)	212 (52.7)	0/(0.0)
MICROBIOLOGY ANALYSIS			
Sputum sample collected, n (%)	321 (33.6)	321 (79.9)	633/(66.3)
Positive culture * samples, n (%)	73 (7.7)	73 (18.2)	0/(0.0)
Blood sample collected, n (%)	813 (87.5)	346 (88.5)	25/(2.6)
Positive blood * culture samples, n (%)	92 (9.6)	25 (6.2)	0/(0.0)
SEVERITY ASSESSMENT			
CURB-65 ** ≥ 3, n (%)	122 (13.0)	53 (13.5)	16/(1.7)
Triage ****			59/(6.2)
Red/orange, n (%)	233 (26.0)	127 (34.2)	
Yellow, n (%)	479 (53.5)	196 (52.8)	
Green/blue, n (%)	183 (20.4)	48 (12.9)	
Fever ≥ 38 °C, n (%)	263 (27.6)	107 (26.6)	0/(0.0)
C-reactive protein			0/(0.0)
Low < 20 mg/L, n (%)	196 (20.5)	74 (18.4)	
Moderate 21–99 mg/L, n (%)	291 (30.5)	138 (34.3)	
High ≥ 100, n (%)	467 (49.0)	190 (47.3)	
ANTIBIOTIC TREATMENT			
Antibiotic allergies			
Penicillin allergy, n (%)	66 (6.9)	30 (7.5)	0/(0.0)
Other antibiotic allergies, n (%)	22 (2.3)	10 (2.5)	0/(0.0)
Antibiotic prescription within one month prior to admission, n (%)	339 (35.5)	131 (32.6)	0/(0.0)
Antibiotic treatment at time of admission, n (%)	259 (27.1)	102 (25.4)	0/(0.0)

* Significant positive results reported to the patient medical records; ** CURB-65: (confusion, uremia, respiratory rate, blood pressure, age > 65 years); **** Danish emergency process triage DEPT [17]; CAP: community-acquired pneumonia.

## Data Availability

Due to Danish laws on personal data, data cannot be shared publicly. To request data, please contact the corresponding author for more information. The person responsible for the research was the principal investigator (CBM) and corresponding author (MBC) in collaboration with the University Hospital of Southern Denmark. This organization owns the data and can provide access to the final dataset.

## Supplementary Materials

The following supporting information can be downloaded at: https://www.mdpi.com/article/10.3390/antibiotics12121680/s1, Figure S1: Clinical guidelines for the management of community-acquired pneumonia based on CURB-65 score < 3; Figure S2: Clinical guidelines for the management of community-acquired pneumonia based on CURB-65 score ≥ 3; Table S1: Antibiotic prescription for community-acquired pneumonia, and the total number of infections.
