# Supplementary material for: Antibiotic Guideline Adherence at the Emergency Department: A Descriptive Study from a Country with a Restrictive Antibiotic Policy
Source: Antibiotics (Basel). 2023 Nov 29;12(12):1680. doi: 10.3390/antibiotics12121680 (PMC10740443; doi:10.3390/antibiotics12121680)

Supplementary material

Title: Antibiotic prescription pattern at the Emergency Department: A descriptive study from a country with low antimicrobial resistance

Authors: Mariana B. Cartuliares [1, 2†], Sara N. Søgaard[1†], Flemming S. Rosenvinge [3, 4], Christian B. Mogensen [1,2], Mathias Amdi Hertz [5,6], Helene Skjøt-Arkil [1,2]

[1] Department of Emergency Medicine, University Hospital of Southern Denmark, , 6200 Aabenraa, Denmark; mbc@rsyd.dk (M.B.C.)

[2] Department of Regional Health Research, University of Southern Denmark, 6200 Aabenraa, Denmark

[3] Odense University Hospital, Department of Clinical Microbiology, 5000 Odense C, Denmark

[4] Research Unit of Clinical Microbiology, Department of Clinical Research, University of Southern Denmark,, 5000 Odense C, Denmark

[5] Department of Infectious Diseases, Odense University Hospital, University of Southern Denmark, 5000 Odense C, Denmark

[6] Research Unit of Infectious Diseases, Department of Clinical Research, University of Southern, 5000 Odense C, Denmark

* Correspondence: Sara.Norgaard.Sogaard2@rsyd.dk

† These authors contributed equally to this work.

Table of content

Figure S1: Clinical guidelines for the management of community-acquired pneumonia based on CURB-65 score < 3.

Figure S2: Clinical guidelines for the management of community-acquired pneumonia based on CURB-65 score ≥ 3.

Table S1: Antibiotic prescription for community-acquired pneumonia, and the total number of infections.

Table S1: Antibiotic prescription for community-acquired pneumonia, and the total number of infections.

Infection focus	CAP* n (%)	Total n (%)
Total	402 (42.1)	954 (100.0)
ANTIBIOTICS AT 4-HOUR		
No	79 (19.7)	226 (23.7)
Oral	77 (19.2)	174 (18.2)
Intravenous	244 (60.7)	545 (57.1)
Combination of oral and intravenous	2 (0.5)	9 (0.9)
Narrow-spectrum penicillins	148 (36.8)	244 (25.6)
Extended-spectrum penicillin	26 (6.5)	104 (10.9)
Penicillin with beta-lactamase inhibitor	122 (30.3)	307 (32.2)
Critical broad-spectrum antibiotics	20 (5.0)	58 (6.1)
Macrolides	35 (8.7)	40 (4.2)
Others	7 (1.7)	62 (6.5)
ANTIBIOTICS AT 48-HOURS		
No	78 (19.4)	205 (21.5)
Oral	138 (34.3)	330 (34.6)
Intravenous	182 (45.3)	410 (43.0)
Combination of oral and intravenous	4 (1.0)	8 (0.8)
Narrow-spectrum penicillins	121 (30.1)	233 (24.4)
Extended-spectrum penicillin	29 (7.2)	129 (13.5)
Penicillin with beta-lactamase inhibitor	136 (33.8)	289 (30.3)
Critical broad-spectrum antibiotics	29 (7.2)	74 (7.8)
Macrolides	19 (4.7)	32 (3.4)
Others	12 (3.0)	46 (4.8)
ANTIBIOTICS ON DAY-5		
No	92 (22.9)	259 (27.2)
Oral	218 (54.4)	494 (51.9)
Intravenous	86 (21.4)	192 (20.2)
Combination of oral and intravenous	5 (1.2)	7 (0.7)
Narrow-spectrum penicillins	104 (25.9)	211 (22.1)
Extended-spectrum penicillin	37 (9.2)	160 (16.8)
Penicillin with beta-lactamase inhibitor	126 (31.3)	218 (22.9)
Critical broad-spectrum antibiotics	23 (5.7)	68 (7.1)
Macrolides	19 (4.7)	27 (2.8)
Others	16 (4.0)	49 (5.1)

All numbers are presented as numbers (n) and percentages (%)
*CAP: community-acquired pneumonia

Figure S1: Clinical guidelines for the management of community-acquired pneumonia based on CURB-65 score < 3.

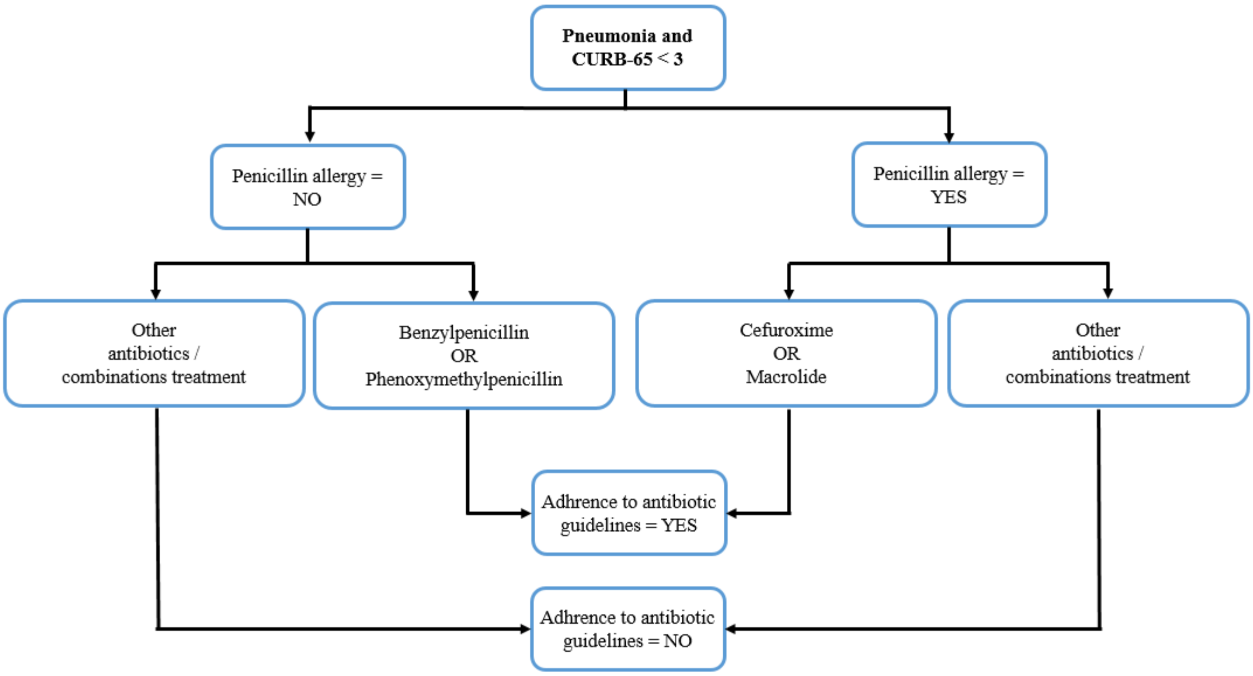

Figure S2: Clinical guidelines for the management of community-acquired pneumonia based on CURB-65 score ≥ 3.

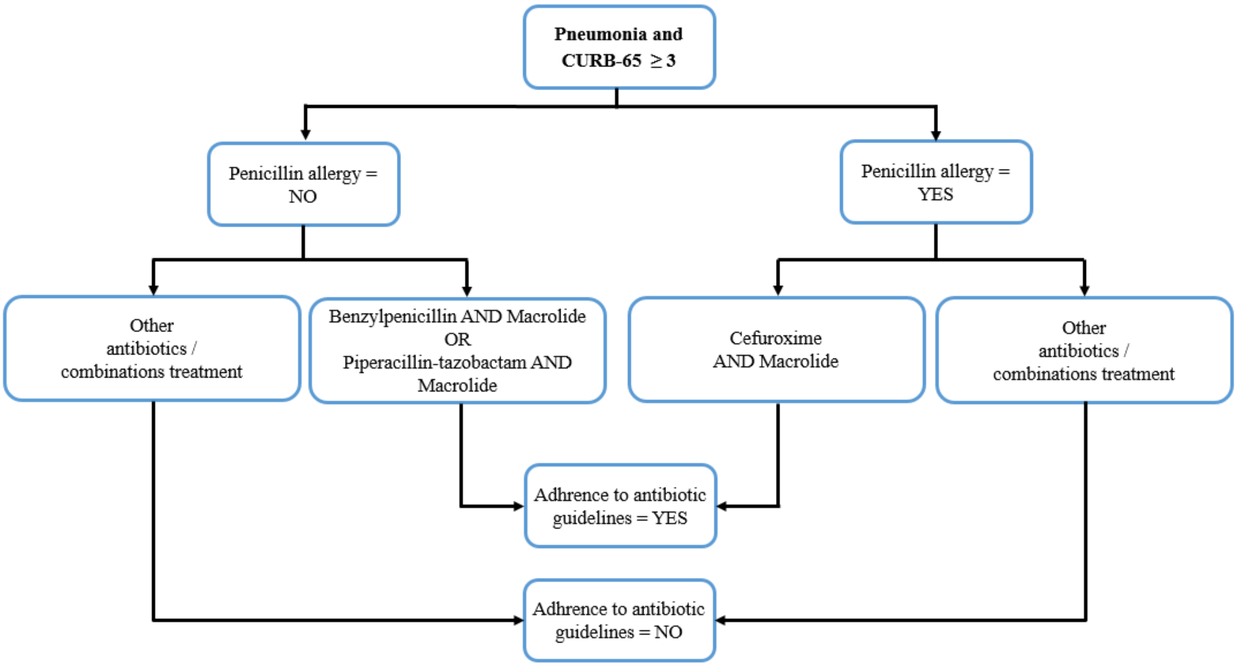

Supplement: Supplementary file 1 [file antibiotics-12-01680-s001.zip › Supplementary material_proof.pdf]
